# Synthesis and characterization of benzodioxinone mono-telechelics and their use in block copolymerization

**DOI:** 10.3906/kim-2104-22

**Published:** 2021-08-27

**Authors:** Cumali ÇELİK

**Affiliations:** 1 Property Protection and Security Department, Yalova Vocational School, Yalova University, Yalova Turkey

**Keywords:** Benzodioxinones, block copolymer, click chemistry, ketene chemistry, photoacylation

## Abstract

Poly(methyl methacrylate) (PMMA) and poly(ethylene glycol) methyl ether (mPEG)-based monotelechelics were quantitatively prepared by copper (I)-catalyzed azide/alkyne cycloaddition (CuAAC) click reactions using azido-terminated polymers and alkyne functional benzodioxinones. The monotelechelic containing dimethyl moities (2,2-dimethyl-5-(prop-2-yn-1-yloxy)-4H-benzo[d][1,3]dioxin-4-one) was heat-sensitive, whereas the monotelechelic containing diphenyl moieties (2,2-diphenyl-5-(prop-2-yn-1-yloxy)-4H-benzo[d][1,3]dioxin-4-one) was UV light sensitive. Based on the FT-IR, ^1^H-NMR, and GPC investigations, the CuAAC click reactions enable the quantitative syntheses of monotelechelics under mild conditions. Moreover, the photosensitive mPEG-based monotelechelic was further utilized for the block copolymer synthesis upon UV-light irradiation. The photoinduced acylation of mPEG monotelechelic consist of (2,2-diphenyl-5-(prop-2-yn-1-yloxy)-4H-benzo[d][1,3]dioxin-4-one) in the presence of hydroxy-terminated poly(epsilon caprolactone) enabled the successful block copolymer formation.

## 1. Introduction

Telechelic polymers are macromolecules consisting of reactive groups at both chain ends are widely used as building blocks for numerous macromolecular architectures including block and graft copolymers and star polymers as well as chain extenders, cross-linkers for polymer networks [1]. In polymer chemistry, telechelic polymers can be classified as mono-telechelic, di-telechelic and tri-telechelic according to their number of reactive end-groups [2]. There are many routes for the preparation of telechelic polymers with terminal reactive functionalities in the literature [3]. At the beginning of polymer chemistry, conventional radical polymerization, polycondensation and ionic polymerization techniques were mostly used for this purpose [1]. After discoveries of controlled radical polymerization (CRP) including nitroxide mediated radical polymerization (NMRP), atom transfer radical polymerization (ATRP) and reversible addition−fragmentation chain-transfer (RAFT) polymerization as well as the click chemistry reactions including copper (I)-catalyzed azide/alkyne cycloaddition (CuAAC), DielsAlder and thiol-yne/ene, it is easy to synthesize telechelic polymers with high yield under mild conditions [49]. For example, several functionalities including -alcohol, -carboxylic acid, -pyrene, and -benzyl moieties have been successfully installed on the terminal position of polystyrene (PSt) and poly(epsilon caprolactone) (PCL) via photoinduced version of CuAAC click chemistry using 2,4,6-trimethylbenzoyl)diphenylphosphine oxide/copper (II) chloride/ N,N,N--′,N″,N″-pentamethyldiethylenetriamine as catalyst [4]. The efficiency of the photoinduced CuAAC click reactions were found higher than 74% that determined by ^1^H-NMR spectroscopy using the ratio of the characteristic peaks of functional groups compared with PSt and PCL.

 Benzodioxinone is an important class of either photo- or heat-sensitive compounds that is responsible to generate the reactive quino-ketene upon heat or UV illuminations. The produced ketenes can be readily reacted with amines, phenols, and alcohols to get related salicylic amide and esters [10,11]. Thermolysis of benzodioxinones at 200 C in the absence of nucleophiles resulted in dimerization reaction, whereas the corresponding salicylic amides or esters were formed in high yields in the presence of nucleophiles such as alcohols or amines [12^o^15]. Compared to the heat-sensitive benzodioxinone, photochemically activated one has several advantages such as being applicable at low temperatures with low-energy consumption and provides spatial and temporal regulation of chemical processes [10,1622]. Furthermore, the liberated benzophenone as a by-product can be further employed as a photoinitiator for both free radical and cationic --photopolymerizations of (meth)acrylates and epoxides. Additionally, various macromolecular structures including block and graft copolymers and polymer networks were simply prepared via benzodioxinone chemistry upon UV irradiation at ambient temperature [10,1624]. In this study, firstly both heat- (2,2-dimethyl-5-(prop-2-yn-1-yloxy)-4h-benzo[d][1,3]dioxin-4-one) and photo-sensitive (2,2-diphenyl-5-(prop-2-yn-1-yloxy)-4h-benzo[d][1,3]dioxin-4-one) benzodioxinones (Alkyne-DPh-Bd and Alkyne-DMe-Bd) were successfully synthesized using modified literature. Then these compounds were easily installed on the terminal position of both azido-functionalized poly(methyl methacrylate) and poly(ethylene glycol) methyl ether in order to get their mono-telechelics via CuAAC click chemistry. The mild UV irradiation of the obtained photosensitive-monotelechelic (Alkyne-DPh-Bd) in the presence of monohydroxyl-functionalized PCL resulted in corresponding block copolymer nearly quantitative yield.

## 2. Experimental

### 2.1. Materials

Poly(ethylene glycol) methyl ether, (mPEG-OH, *M*_n_ = 5000 g/mol, Aldrich, Darmstadt, Germany), dimethoxyethane (%99.5, Fluka), 4-dimethylaminopyridine (DMAP, 99%, Acros, Geel, Belgium), thionyl chloride (%99.5 Acros), benzophenone (99%, Aldrich), acetone (%99,5 Merck, Darmstadt, Germany) was used as received. Methyl methacrylate (MMA, 99%, Aldrich) was purified via filtering over basic Al_2_O_3_. *N,N,N* ′ *,N* ″ *,N* ″ *-* pentamethyldiethylenetriamine (PMDETA, 99%, Aldrich) was distilled before use. Epsilon-caprolactone (eCL, Aldrich, 97 %) was vacuum distilled over calcium hydride. Copper(I) bromide (98%, Acros), ethyl 2-bromopropionate (99%, Aldrich), methyl chloride, triethylamine (%99,5 Sigma-Aldrich), sodium azide (NaN_3_, 99,5%, Fischer Scientific, Pittsburgh, USA), L-ascorbic acid sodium salt (NaAsc, Acros 99%), copper(II)sulfate.5H_2_O (Cu(II)SO_4_, Acros 99%), tin(II) 2-ethyl-hexanoate (Sn(Oct)_2_, Aldrich, 95%) and n-propanol (n-PA, Aldrich, 99.77%) were used as received. Tetrahydrofuran (THF, >99,8%), anhydrous dichloromethane (>99,8%), dimethylformamide (DMF, 99,8%), toluene (99,8%) was purchased from Sigma-Aldrich. Hexane, methanol, and brine were technical grades. Silica gel (Al_2_SO_3,_ Merck, 0.0400.063 mm) and Na-_2_SO_4 _(ACS reagent, ≥99.0%, anhydrous, powder) were used for purification steps of PEG-N_3_ and MMA.

### 2.2. Synthesis of azido-functionalized poly(ethylene glycol) methyl ether (mPEG-N3)

#### 2.2.1. Mesillation of mPEG

mPEG5000 (2 g, 0.4 × 10^−3^ mol -OH group) was dissolved at 30 mL dry dichloromethane in 50 mL round bottom flask. Subsequently, triethylamine (0.56 mL, 4 × 10^−3^ mol) was added to the solution as a catalyst. After that, methylsulfonyl chloride was added drop by drop to solution a inert atmosphere at 0°C. The was stirred vigorously gradually ris to room temperature for 24 h. The reaction solution was precipitated in cold diethyl ether (100 m) and filtered via suction filtration. The filtrate was dried in a vacuum oven at room temperature for 24 h. The yield was found as 1.76 g and calculated as 88%, gravimetrically.

nreaction atingl FT-IR (υ_max_): 2886 and 2613 cm^1^^-^ (S-C stretching), 2494 and 1171 cm^1 ^^-^(-SO_2_ stretching). 

#### 2.2.2. Azidation of mPEG-MSCl

Obtained mesylated mPEG_5000_ (1g, 0.2 × 10^−3^ mol) was dissolved in DMF (15 mL) followed by excess sodium azide (0.13 g, 2 × 10^−3^ mol) addition. The solution was heated up to 45°C and stirred for 3 days. Dry dichloromethane (30 mL) was added to the solution and washed with distilled water 3 times. The organic phase was separated by 250 mL separation funnel and dried over Na_2_SO_4_ and filtered. The obtained solution was concentrated to 30 mL by rotary evaporator and precipitated with diethyl ether (300 mL), filtered, and dried in vacuum oven for 24h. The yield (0.92 g, 92%, gravimetrically) and molecular characteristics of PMMA-N the mixture_3_ were found as *M*_n,GPC _= 5100 g/mol and *Ɖ* = 1.41.

FT-IR (υ_max_): 3401 cm^1^^-^ (OH), 2920 cm^1^^-^ (C–H), 2111 cm^1^^-^ (N_3_).

### 2.3. Synthesis of azido-functionalized poly (methyl methacrylate) (PMMA-N3)

Firstly, a bromo-functionalized poly(methyl methacrylate) (PMMA-Br) was synthesized by ATRP according to the published procedure [25]. The yield and molecular characteristics of PMMA-Br were found as M_n,GPC _= 3700 g/mol and *Ɖ* = 1.33. And then, the obtained PMMA-Br (2 g, 5.12 × 10^−3^ mol) and sodium azide (0.167 g, 21.6 × 10^−3^ mol) were dissolved in 40 mL DMF in a 100 mL round bottom flask. The reaction mixture was deaerated by nitrogen and stirred via a magnetic stirrer at 60°C for overnight. After the given time, the mixture was cooled to room temperature and precipitated in 400 mL technical grade methanol. The precipitate was filtrated via a suction filtration setup and dried in a vacuum oven. The yield (1.88 g, 94%, gravimetrically) and molecular characteristics of PMMA-N_3_ were found as *M*_n,GPC _= 3900 g/mol and *Ɖ* = 1.37.

FT-IR (υ_max_): 2990 cm^1^^-^ (C-H, alkyl), 2950 cm^1^^-^ (C-H, alkyl), 2100 cm^1^^-^ (N_3_), 1730 cm^1^^-^ (C=O).

### 2.4. Synthesis of hydroxyl-functionalized poly(epsilon caprolactone) (PCL-OH)

The PCL-OH was synthesized by ring-opening polymerization followed by the published procedure [26]. The eCL (5.0 mL, 45.12 mmol) was added as a monomer in Schlenk tubes equipped with a magnetic stirrer and then a solution of tin(II) 2-ethyl-hexanoate in toluene (1.0 mL, 2.256 × 10^2^^-^ mmol), a solution of n-propanol in toluene (1.0 mL, 0.45 mmol) and toluene (3.0 mL) were added under nitrogen. The ring-opening polymerization of eCL was carried out at 110 C for 24 h. After the given time, the mixture was poured into a 10-fold excess of cold heptane. The resulting hydroxyl-functionalized PCL was filtrated and dried at room temperature in a vacuum oven and obtained as a white powder. The yield (3.35 g and 65%, gravimetrically) and molecular characteristics of PCL-OH were found as ^o^ *M*_n,GPC _= 7400 g/mol and *Ɖ* = 1.34. 

FT-IR (υ_max_): 2950 cm^1 ^^-^(asymmetric C-H stretching), 2870 cm^1^^-^ (symmetric C-H stretching), 1725 cm^1^^-^ (C=O stretching), 1295 cm^1 ^^-^(C–O and C–C stretching), 1240 cm^1^^-^ (asymmetric C–O–C, stretching), and 1170 cm^1 ^^-^(symmetric C–O–C stretching).

^1^H-NMR (CDCl_3_, 500 MHz,): 4.00 (CH_2_O on PCL), 3.65 (2H, CH_2_OH), 3.45 (CH_2_-CH_2_-CH_3_), 2.50 (1H, CH_2_–C≡CH), 2.45–2.41 (CH_2_-CH_2_-CH_3_), 2.35–2.27 (CH_2_C=O on PCL), 1.67–1.57 (CH_2_ on PCL and CH_2_-CH_2_-CH_3_), 1.40-1.38 (CH_2 _on PCL).

### 2.5. Synthesis of 2,2-Diphenyl-5-(Prop-2-yn-1-yloxy)-4H-Benzo[d][1,3]Dioxin-4-one (Alkyne-DPh-Bd) and 2,2-Dimethyl-5-(Prop-2-yn-1-yloxy)-4H-Benzo[d][1,3]Dioxin-4-one (Alkyne-DMe-Bd)

**Scheme 1:**
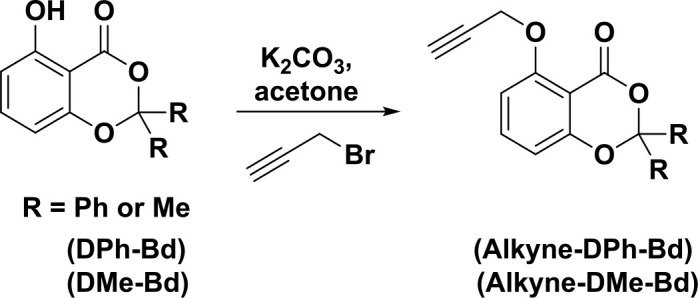
Synthesis procedures of Alkyne-DPh-Bd and Alkyne-DMe-Bd.

The precursor, 5-hydroxy-2,2-diphenyl-4h-benzo[d][1,3]dioxin-4-one (DPh-Bd) was synthesized by following published procedure (Scheme 1) [27,28]. The resulting DPh-Bd compound (1 g, 3.2 × 10^−3^ mol) and anhydrous potassium carbonate (4.4 g, 32 × 10^−3^ mol) was dissolved in acetone (30 mL) and then stirred for 2 h at room temperature. After the given time, the propargyl bromide (1 mL, 10 × 10^−3^ mol) was first added into the reaction mixture that was subsequently heated at 56 °C for 4 h. In the final stage, the acetone was removed by a rotary evaporatorto and then diethyl ether was added into the remaining mixture, washed with water three times after that brine, and then the organic phase was dried over anhydrous sodium sulphate. The remaining mixture was purified by a silica column chromatography using n-hexane/ethyl acetate (90/10) as the eluent. The Alkyne-DPh-Bd was obtained as a light yellow solid (0.8 g, 70%, mp = 163 °C). 

,FT-IR (υ_max_): 3400 cm^1^^-^ (OH), 2948 cm^1^^-^ (C-H, Ar), 2800 cm^1^^-^ (C-H, alkyl), 2100 cm^1^^-^ (C≡C), 1728 cm^1^^-^ (C=O), 1608 cm^1^^-^ (C-C, Ar), 1460 cm^1 ^^-^(C-C, Ar), 1100 cm^1^^-^ (C-O), 705 (C-H, Ar) cm^-1^. 

^1^H-NMR (CDCl_3_, 500 MHz,): δ = 7.60-7.2 (m, 10H, Ar), 6.7-6.5 (d, 3H, Ar), 4.9 (s, 2H, OCH_2_), 2.5 (s, 1H, C≡CH). 

The 5-hydroxy-2,2-dimethyl-4h-benzo[d][1,3]dioxin-4-one (DMe-Bd) compound was also prepared by a similar procedure of DPh-Bd using acetone instead of benzophenone. Rest of the synthesis procedure for Alkyne-DMe-Bd was same with above. The Alkyne-DMe-Bd was obtained as a light yellow solid (1.15 g, 64%, mp = 142 °C).

FT-IR (υ_max_): 3410 cm^1^^-^ (OH), 2946 cm^1^^-^ (C-H, Ar), 2805 cm^1^^-^ (C-H, alkyl), 2103 cm^1^^-^ (C≡C), 1730 cm^1^^-^ (C=O), 1605 cm^-1^ (C-C, Ar), 1458 cm^1 ^^-^(C-C, Ar), 1115 cm^1^^-^ (C-O), 715 (C-H, Ar) cm^1^^-^. 

^1^H-NMR (CDCl_3_, 500 MHz,): δ = 6.7-6.5 (d, 3H, Ar), 4.9 (s, 2H, OCH_2_), 2.5 (s, 1H, C≡CH), 1.7 (s, 6H, CH_3_).

### 2.6. Synthesis of mPEG-based Benzodioxinone ono-telechelics via CuAAC lick eactionMCR

**Scheme 2:**
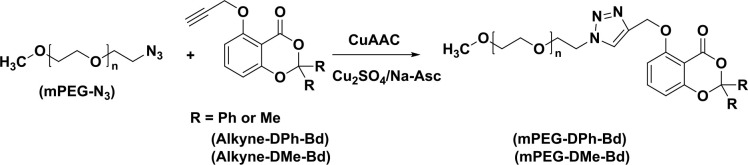
Synthesis procedures of mPEG-DPh-Bd and mPEG-DMe-Bd.

The mPEG-N_3_ (0.51 g, 0.1 × 10^−3^ mol) and Alkyne-DPh-Bd (0.0356 g, 0.1 × 10^−3^ mol) was dissolved in 10 mL dissolved. Then, Cu_2_SO_4_.5H_2_O (0.025 g, 0.1 × 10^−3^ mol) and sodium ascorbate (0.02 g, 0.1 × 10^−3^ mol) were added to the solution and vigorously stirred for overnight at room temperature. After the given time, the mixture was precipitated in hexane, filtered, and dried in a vacuum oven at room temperature. The click reaction of PEG-N_3 _and Alkyne-DMe-Bd was carried out with the same procedure (Scheme 2). 

The yield (0.50 g, 92%, gravimetrically) and molecular characteristics of mPEG-DPh-Bd were found as *M*_n,GPC _= 5300 g/mol and and *Ɖ* = 1.39.

The yield (0.45 g, 85%, gravimetrically) and molecular characteristics of mPEG-DMe-Bd were found as *M*_n,GPC _= 5200 g/mol and and *Ɖ* = 1.36.

### 2.7. Synthesis of PMMA-based benzodioxinone mono-telechelics via CuAAC click reaction

**Scheme 3:**
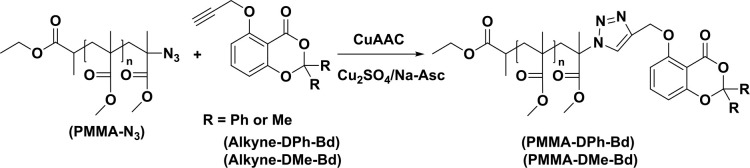
Synthesis procedures for PMMA-DPh-Bd and PMMA-DMe-Bd.

The obtained PMMA-N_3_ (0.68 g, 0.2 × 10^−3^ mol) and Alkyne-DPh-Bd (0.071 g, 0.2 × 10^−3^ mol) were dissolved in 10 mL dichloromethane into 25 mL round bottom flask. Then, Cu_2_SO_4_.5 H_2_O (0.032 g, 1.28 × 10^−3^ mol) and Na Ascorbate (0.025 g, 1.28 × 10^−3^ mol) were added to solution and vigorously stirred at room temperature overnight. the reaction was precipitated in methanol, filtered, and dried in a vacuum oven. The click reaction of PMMA-NAfter _3 _and Alkyne-DMe-Bd was carried out with the same procedure (Scheme 3). 

The yield (0.71 g, 94%, gravimetrically) and molecular characteristics of PMMA-DPh-Bd were found as *M*_n,GPC _= 4400 g/mol and *Ɖ* and = 1.31.

The yield (0.65 g, 89%, gravimetrically) and molecular characteristics of PMMA-DMe-Bd were found as *M*_n,GPC _= 4200 g/mol and and *Ɖ* = 1.28.

### 2.8. Synthesis of mPEG-b-PCL copolymer via photoinduced ketene chemistry

**Scheme 4:**
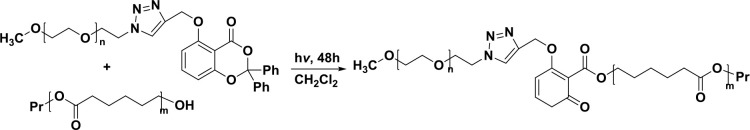
Synthesis procedure for mPEG-b-PCL copolymer via photoinduced ketene chemistry.

The mPEG-DPh-Bd (0.102 g, 0.02 × 10^−3^ mol) and PCL-OH (0.148 g, 0.02 × 10^−3^ mol) were dissolved a small amount of CH_2_Cl_2_ (1 mL) in a quartz tube. Subsequently, the reaction mixture was degassed with nitrogen and was placed in a UV photoreactor to illuminate UV light-emitting 370 nm for 48 h (Scheme 4). Finally, the product was precipitated in cold methanol and dried in a vacuum at room temperature after filtration. The yield (0.203 g, 81%, gravimetrically) and molecular characteristics of PCL-OH were found as *M*_n,GPC _= 10800 g/mol and *Ɖ* = 1.44.

### 2.9. Characterization

Fourier transform infrared (FT-IR) and ^1^H-NMR spectra of the intermediates and final polymers were recorded on a Perkin-Elmer (Perkin Elmer Italia S.p.A., Milano, Italy) FT-IR Spectrum One B and Varian Unity Inova 500 MHz spectrometers, respectively. The ^1^H-NMR measurements were performed in CDCl_3_ with Si(CH_3_)_4_ as the internal standard at room temperature. Molecular characteristics (molecular weights and molecular weight distributions of resulting polymers were determined by size exclusion chromatography (SEC) by a Viscotek GPCmax Autosampler consisting of a Viscotek differential refractive index detector, a pump, three ViscoGEL GPC columns (G4000H_HR_, G3000H_HR_ and G2000H_HR_) with a tetrahydrofuran flow rate of 1.0 mL/min at 30 °C. The refractive index detector was calibrated by a series of polystyrene standards which had a narrow polydispersity set consisting of 7 individual standards. All SEC data were analyzed using Viscotek (Malvern Panalytical Ltd., Malvern, UK) OmniSEC Omni–01 software. (Malvern Panalytical Ltd., Malvern, UK)

## 3. Results and Discussion

Ketenes are very reactive molecules towards unsaturated and nucleophilic compounds. The reactions of ketenes with amines, alcohols, and carboxylic acids are simple routes for the synthesis of corresponding amides, esters, and anhydrides. However, all ketene derivatives are unstable compounds that cannot be isolated due to their instabilities. Therefore, the desired ketenes can be in-situ generated by either thermolysis or photolysis of precursors and readily reacted with antagonist nucleophilic compounds. Benzodioxinones are important precursors to form reactive ketenes upon heat or UV illuminations. Recently, benzodioxinone chemistry has been utilized in homopolymerization, block, and graft copolymerization and cross-linking reactions for the construction of various macromolecular architectures including homopolymer block copolymers, graft copolymers and polymer networks. Here, thermally (Alkyne-DMe-Bd) and photochemically (Alkyne-DPh-Bd) active alkyne functionalized benzodioxinones were firstly synthesized by etherification reactions of propargyl bromide with either 5-hydroxy-2,2-dimethyl-4h-benzo[d] [1,3]dioxin-4-one or 5-hydroxy-2,2-diphenyl-4h-benzo[d][1,3]dioxin-4-one. Then chemical structures of Alkyne-DMe-Bd and Alkyne-DPh-Bd were confirmed by both FT-IR and ^1^H-NMR spectroscopies. The aromatic and aliphatic C-H bands as well as the C=O band and C-O-C bands at 2950, 2800, 1730, and 1100 cm^-1^ in FT-IR spectroscopy confirmed the chemical structures of Alkyne-DMe-Bd and Alkyne-DPh-Bd. In addition, the characteristic aromatic bands of benzodioxinone and alkyne groups in both Alkyne-DMe-Bd and Alkyne-DPh-Bd molecules were clearly detected at 6.76.5, 4.9 and 2.5 ppm, respectively. Furthermore, the phenyl groups of Alkyne-DPh-Bd coming from benzophenone and methyl groups of Alkyne-DMe-Bd coming from acetone were also revealed at 7.607.2 and 1.7 ppm (see experimental part).

**Figure 1:**
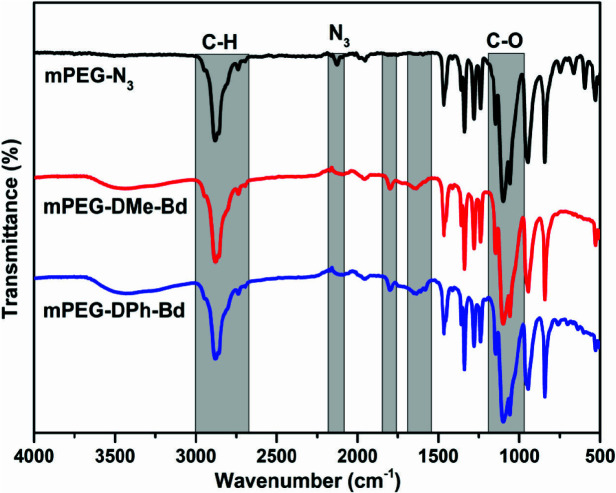
FT-IR spectra of mPEG-N3, mPEG-DPh-Bd and mPEG-DMe-Bd.

After successful synthesis of initial Alkyne-DPh-Bd and Alkyne-DMe-Bd, their installations on the azido groups of mPEG-N_3_ were done by CuAAC click reactions at room temperature using Cu_2_SO_4_/sodium ascorbate catalyst system. For both cases, quantitative yields were determined gravimetrically (92 and 85% for mPEG-DPh-Bd and mPEG-DMe-Bd, respectively). On the other hand, the CuAAC click reactions were monitored by FT-IR spectroscopy through the disappearance of the characteristic azido band at 2111 cm^1^^-^. The complete disappearance of azido peak in the FT-IR spectra of mPEG-DPh-Bd and mPEG-DMe-Bd in Figure 1 proved the successful CuAAC click reactions.

In addition to FT-IR investigations, the Figure 1.

**Figure 2:**
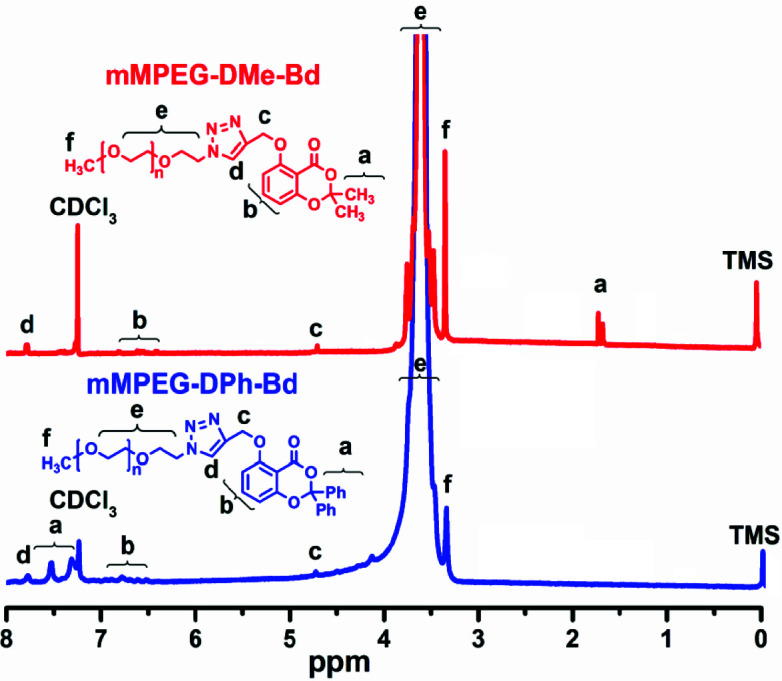
^1^H-NMR spectra of mPEG-N_3_, mPEG-DPh-Bd and mPEG-DMe-Bd.

^1^H-NMR spectroscopy gives more detailed information about the chemical structures of mPEG-DPh-Bd and mPEG-DMe-Bd monotelechelics (Figure 2). In both cases, the characteristic peaks (e+f) of mPEG were clearly detected around 3.313.92 ppm, while the aromatic and aliphatic peaks of benzodioxinones also appeared at 6.517.63, 1.73 and 4.76 ppm. Furthermore, the new peak corresponding to triazole rings at 7.83 ppm confirmed the successful installation of benzodioxinones onto the terminal positions of mPEG. On the other hand, the molecular weights of the resulting polymers were slightly higher than mPEG-N-_3_ due to contributions of Alkyne-DPh-Bd and Alkyne-DMe-Bd molecules. On the contrary, the polydispersity indices of the mPEG-DPh-Bd and mPEG-DMe-Bd monotelechelics were negligibly decreased from 1.41 to 1.39 and 1.36. This could be due to the elimination of possible unfunctionalized mPEG chains after the solution/precipitation procedure. 

Finally, the peak areas of the aliphatic protons of mPEG (e and f) and aromatic protons of benzodioxinones (b) in ^1^H-NMR spectra of mPEG-DPh-Bd and mPEG-DMe-Bd monotelechelics were utilized to calculate their molecular weights. The *M*_n,NMR_ values of mPEG-DPh-Bd and mPEG-DMe-Bd were found as 5200 and 5100 g/mol, respectively.

**Figure 3:**
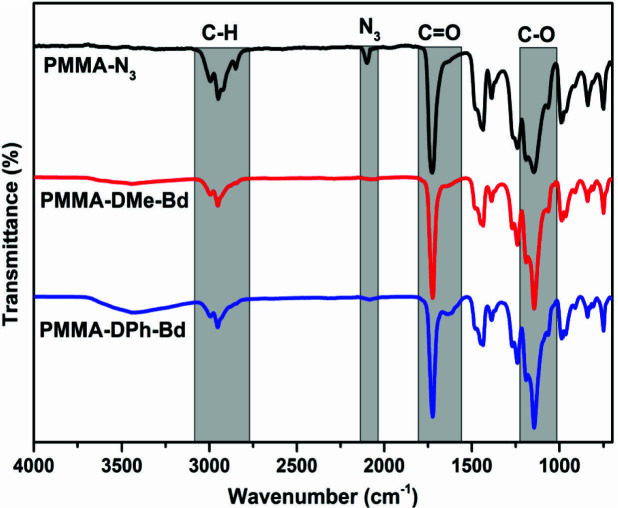
FT-IR spectra of PMMA-N_3_, PMMA-DPh-Bd and PMMA-DMe-Bd.

The similar CuAAC click chemistry was also applied for the functionalization of PMMA-N_3_ with Alkyne-DPh-Bd and Alkyne-DMe-Bd. The chemical structures of PMMA-DPh-Bd and PMMA-DMe-Bd were confirmed by FT-IR and ^1^H-NMR spectroscopy analysis. The complete disappearance of azido band at 2100 cm^1^^-^ of PMMA-N_3_ approved the successful syntheses of PMMA-DPh-Bd and PMMA-DMe-Bd in Figure 3. Moreover, the characteristic C=O, C-O-C, and aromatic bands of both PMMA and benzodioxinones units also determined FT-IR spectra of PMMA-DPh-Bd and PMMA-DMe-Bd.

**Figure 4:**
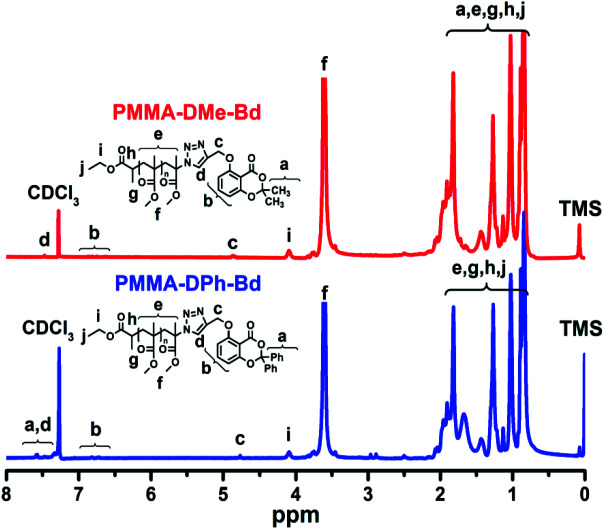
^1^H-NMR spectra of PMMA-N_3_, PMMA-DPh-Bd and PMMA-DMe-Bd.

The were ^1^H-NMR analyses of PMMA-DPh-Bd and PMMA-DMe-Bd were accomplished and the characteristic bands of both PMMA and benzodioxinones components were assigned in Figure 4. The aromatic and aliphatic groups of benzodioxinones were confirmed by detecting a, b, and c peaks at 7.61, 6.62 and 4.83 ppm, respectively. In addition, the aliphatic protons coming from ATRP initiator (g+hi+j) and PMMA (e+f) also appeared at 0.782.05, 3.69 and 4.07 ppm. The peak areas of the aliphatic protons of PMMA (f) and aromatic protons of benzodioxinones (b) in -^1^H-NMR spectra of PMMA-DPh-Bd and PMMA-DMe-Bd monotelechelics were also utilized to calculate their molecular weights. The *M*_n,NMR_ values of PMMA-DPh-Bd and PMMA-DMe-Bd were found as 4250 and 4050 g/mol, respectively.

**Figure 5:**
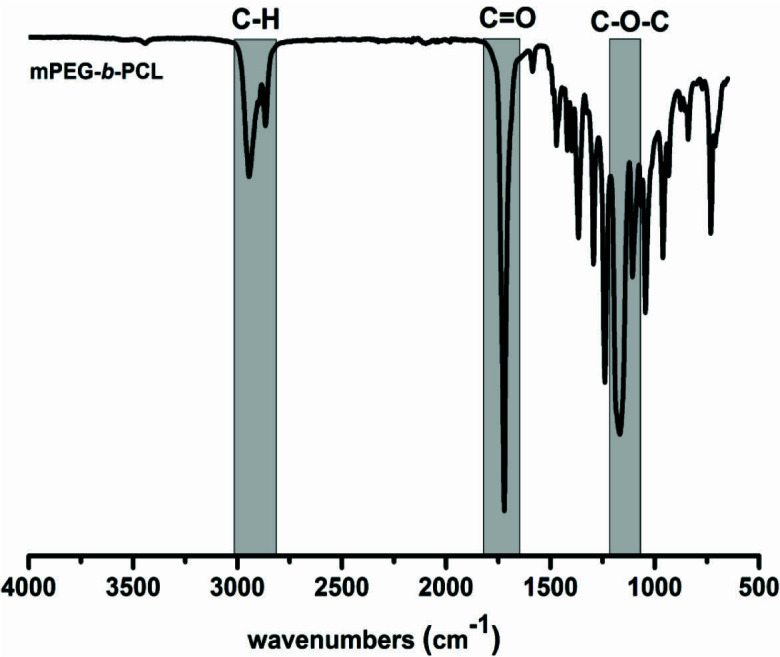
FT-IR spectrum of mPEG-b-PCL.

A model study was also completed to investigate the potential use of photochemically active monotelechelic in the synthesis of block copolymers. The UV exposure of reactive benzodioxinone terminated mPEG-DPh-Bd and antagonist PCL-OH in a UV photoreactor with light-emitting 370 nm for 48 h led to the quantitative block copolymer formation (mPEG- *b* -PCL). Based on the FT-IR spectrum of mPEG- *b* -PCL, the characteristic ester bands of PCL and ether bands of mPEG were clearly detected in Figure 5. This finding confirmed that the obtained mPEG- *b* -PCL consists of both PCL and mPEG blocks.

**Figure 6:**
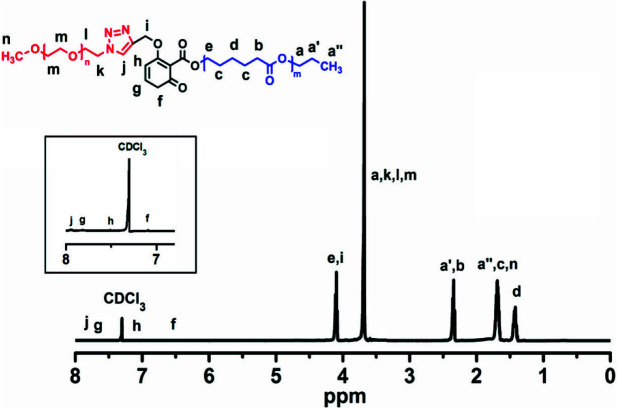
^1^H-NMR spectrum of mPEG-b-PCL.

On the other hand, the distinguished -CH_2_- bands of PCL and -CH_2_- bands of mPEG also appeared at 3.65 and 4.11 ppm in the ^1^H-NMR spectrum of mPEG- *b* -PCL. Moreover, the characteristic triazole (j) and benzodioxinone (f+g+h) bands appeared between 6.5 and 8.0 ppm (Figure 6). The peak areas of the CH_2_ protons of mPEG (m) and O-CH_2_- protons of PCL (e) were utilized to calculate the composition of block copolymers. The composition of mPEG for block copolymer was calculated as 40.1%.

**Figure 7:**
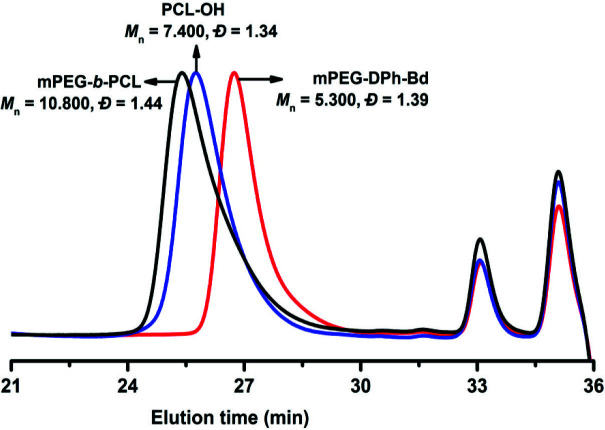
GPC chromatograms of mPEG-DPh-Bd, PCL-OH and mPEG-b-PCL.

In a further investigation, the photoinduced ketene chemistry between mPEG-DPh-Bd and PCL-OH was followed by GPC analyses and their traces were shown in Figure 7. The GPC traces showed us the mPEG-Figure 6. 

*b* -PCL displayed a unimodal and symmetric eluent peak with a slightly broader molecular weight distribution ( *Ð* = 1.44) than mPEG-N_3_ ( *Ð* = 1.41) and mPEG-DPh-Bd ( *Ð* = 1.39). Furthermore, the GPC chromatogram also revealed a significant increase between molecular weights of precursors and block copolymers as they were clearly shifted from 5.300 (mPEG-DPh-Bd) and 7.400 (PCL-OH) to 10.800 (mPEG- *b* -PCL) g/mol. The combined ^1^H-NMR and GPC results clearly confirm that the successful formation of block copolymer without detectable unreactive free precursors. The molecular weights and molecular weight distributions of all precursors, monotelechelics, and block copolymer were summarized in Table 1.

**Table 1:** Summary of data obtained from monotelechelics and its block copolymer.

Entry	Yielda(%)	Mn,GPC(g/mol)	Ɖ	Mn,NMRb(g/mol)	Click efficiencyb(%)
mPEG-N3	92	5100	1.41	4900	-
PMMA-N3	94	3900	1.37	3800	-
PCL-OH	65	7400	1.34	7250	-
mPEG-DPh-Bd	92	5300	1.39	5200	96
mPEG-DMe-Bd	85	5200	1.36	5100	91
PMMA-DPh-Bd	94	4400	1.31	4250	97
PMMA-DMe-Bd	89	4200	1.28	4050	94
mPEG-b-PCL	81	10800	1.44	10300	88

adetermined by gravimetric analysis, bcalculated by 1H-NMR spectroscopy using characteristic peaks of each segments.

## 4. Conclusion

In summary, heat- and light-sensitive monotelechelics were successfully synthesized by CuAAC click chemistry using mPEG-N_3_ and PMMA-N_3_ with Alkyne- DPh-Bd and Alkyne- DMe-Bd compounds under mild conditions. The chemical structures of precursors and monotelechelics were clearly confirmed by spectral analyses using FT-IR and ^1^H-NMR spectroscopies. According the ^1^H-NMR analysis, the desired monotelechelics were quantitatively obtained over 91% CuAAC click efficiency. The photosensitive mPEG-DPh-Bd monotelechelic was further utilized for block copolymer synthesis with antagonist PCL-OH under mild conditions. The ^1^H-NMR spectroscopy and GPC analysis revealed that successful block copolymer formation was achieved upon UV irradiation for 48 h. Consequently, these reactive monotelechelics will utilize vital role for the synthesis of various macromolecular architectures such as block and graft copolymers either heat or UV-light exposure.be d
